# Why and where?—Delay in Tuberculosis care cascade: A cross-sectional assessment in two Indian states, Jharkhand, Gujarat

**DOI:** 10.3389/fpubh.2023.1015024

**Published:** 2023-01-27

**Authors:** Sandul Yasobant, Harsh Shah, Priya Bhavsar, Jay Patel, Somen Saha, Anish Sinha, Tapasvi Puwar, Yogesh Patel, Deepak Saxena

**Affiliations:** ^1^Department of Public Health Science, Indian Institute of Public Health Gandhinagar (IIPHG), Gandhinagar, India; ^2^School of Epidemiology and Public Health, Jawaharlal Nehru Medical College, Datta Meghe Institute of Medical Sciences (Deemed to be University), Wardha, India; ^3^World Health Partners (WHP), Noida, India

**Keywords:** delay, care cascade, tuberculosis, factors for delay, India

## Abstract

Tuberculosis (TB) is the second leading cause of death due to infectious diseases globally, and delay in the TB care cascade is reported as one of the major challenges in achieving the goals of the TB control programs. The main aim of this study was to investigate the delay and responsible factors for the delay in the various phases of care cascade among TB patients in two Indian states, Jharkhand and Gujarat. This cross-sectional study was conducted among 990 TB patients from the selected tuberculosis units (TUs) of two states. This study adopted a mixed-method approach for the data collection. The study targeted a diverse profile of TB patients, such as drug-sensitive TB (DSTB), drug resistance TB (DRTB), pediatric TB, and extra-pulmonary TB. It included both public and private sector patients. The study findings suggested that about 41% of pulmonary and 51% of extra-pulmonary patients reported total delay. Delay in initial formal consultation is most common, followed by a delay in diagnosis and treatment initiation in pulmonary patients. While in extra-pulmonary patients, delay in treatment initiation is most common, followed by the diagnosis and first formal consultation. DR-TB patients are more prone to total delay and delay in the treatment initiation among pulmonary patients. Addiction, co-morbidity and awareness regarding monetary benefits available for TB patients contribute significantly to the total delay among pulmonary TB patients. There were system-side factors like inadequacy in active case findings, poor infrastructure, improper adverse drug reaction management and follow-up, resulting in delays in the TB care cascade in different phases. Thus, the multi-disciplinary strategies covering the gambit of both system and demand side attributes are recommended to minimize the delays in the TB care cascade.

## 1. Introduction

Tuberculosis (TB) is one of the global public health concerns as TB is the second leading cause of death due to infectious diseases (1). In 2020, 1.5 million people lost their lives due to TB worldwide, and India accounted for almost 34% of the total deaths (2). Early care-seeking, accurate and timely diagnosis and prompt treatment initiation are foremost for breaking the transmission chain and effectively controlling TB (3, 4). Delay in care-seeking at any stage of the care cascade among TB patients remains one of the challenges in achieving the intended outcomes of the TB control programs (5–7).

Delays in various phases of the TB care cascade, like the first formal consultation, diagnosis, and treatment, could increase the risk of unfavorable outcomes (including aggravation of the illness and infection, leading to complications, and raising the risk of mortality). And also result in the spreading of TB infection at the community level (8, 9). Reducing the time interval between the onset of symptoms and care-seeking can reduce the incidence of TB as it reduces the window during which infected people with symptoms can spread the disease to others. Evidence suggests that if the average care-seeking delay was reduced by 25% amongst TB patients, TB mortality would be reduced by nearly 6%, and the incidence would decline comparably (10). Therefore, to reduce the overall disease burden and for the significant effect of TB control programs, it is necessary to address the delays amongst TB patients on priority.

The interval for diagnosis and initiation of treatment is higher in high-burden countries and is attributed to both patient and health system-related factors (11, 12). In India, estimated patient, diagnostic, and treatment delays were 18.4 (14.3–27.0), 31.0 (24.5–35.4), and 2.5 days (1.9-3.6), respectively, for TB and chest symptomatic patients combined and the median total delay was 55.3 days (46.5–61.5) (13). The socio-economic condition, disease knowledge and awareness, myths/beliefs, family and social support, and addictions among the patients were reflected in many studies as the prime patient side factors responsible for delay at various phases of the care cascade (14–19).

There are numerous studies on predictors of delay amongst TB patients in India in various phases of the care cascade (13). However, there is a dearth of literature available for these two selected states. The results from other states have been mixed and are more context-specific or study-setting specific (13). Therefore, this study aims to investigate the delay and responsible factors for the delay in the various phases of care cascade among TB patients in two Indian states, Jharkhand and Gujarat. Hence, this study provides a better understanding of the reasons for delays in various phases of care cascade among TB patients for this selected study site. The study findings may aid in designing effective and sustainable interventions to fulfill the gaps in the TB care cascade under the national elimination programme (NTEP).

## 2. Methods

The cross-sectional assessment was carried out from Jan-July 2021 in the two Indian states, Jharkhand and Gujarat.

### 2.1. Study setting

The assessment was done in 10 out of 25 Tuberculosis Units (TUs) in the Purbi Singhbhum and Ranchi districts (Jharkhand state) and 22 out of the 32 TUs in Gandhinagar and Surat districts (Gujarat state). The two states and two districts from each state were selected purposively based on the consultation with the state officials. TU was considered the sampling unit in the demand side, where the probability proportional to size (PPS) sampling follow the simple random sampling. The PPS sampling strategy was used to select the sampling units concerning the patient load and the distribution of the functional TU. A total of 32 TUs were sampled out of 57 TUs based on a random selection with the principle of PPS.

### 2.2. Study design

This study adopts an embedded mixed-method design (Quantitative assessment followed by qualitative interviews) (20) for data collection. The quantitative (cross-sectional survey of TB patients) was carried out, followed by the qualitative [in-depth interview (IDI) both from the system-side actors and demand-side TB patients]. The qualitative interviews were aimed to supplement the larger quantitative TB patient survey.

### 2.3. Study samples and sampling

#### 2.3.1. Quantitative assessment

The quantitative assessment targeted a diverse profile of TB patients, such as drug-sensitive TB (DSTB), drug resistance TB (DRTB), pediatric TB, and extra-pulmonary TB. It included both public and private sector patients. A multistage cluster sampling was used to recruit the patients. A PPS was used to identify the primary sampling unit, TU. From each TU, patients were recruited randomly depending on their availability and willingness to participate. The assessment was intended to cover all stages of the TB care cascade and the delays in the respective phases. Notified TB patients were recruited from 1Q-2019 to 4Q-2020 for DS-TB. Similarly, for DRTB, patients were recruited from 3Q-2018 to 4Q-2020. Patients were interviewed at their convenience, either at the hospital or at their residences. About 990 patients with different categories were recruited for the quantitative assessment. Based on the limited evidence from India on the proportion of delay in the TB care cascade (21) and based on the formative research experiences in the selected study sites, it has been averaged out that 50% of the TB patients delayed seeking care. Therefore, with 95% CI, 90% power, 50% delay, the estimated sample size for the one-sample proportion test (Wald z test) was 778. Adding a non-response rate of an average of 10%, the final sample size for the study was 856.

#### 2.3.2. Qualitative assessment

IDIs were conducted both among the actors from the system side and selected TB patients. About 40 system side actors and 10 TB patients were interviewed to supplement the quantitative findings. The patients and system side actors were interviewed based on their availability and consent for participation. Among the system-side actors, there were healthcare staff from the NTEP programs and the general health system working at the State TB cell, District TB Center, TB Unit, PHI. These actors who were involved in direct contact with TB patient care and decision-making were purposively selected. For the demand side, conveniently, 10 patients were selected at the end of the quantitative assessment to supplement the findings. It was limited to only 10 diverse cases of TB patients and didn't extend beyond till the saturation of the responses due to the project timeline.

### 2.4. Data collection

The quantitative data were collected through a structured delay assessment questionnaire, and the semi-structured interview guide was prepared for qualitative assessment in vernacular language. Trained researchers were administered the survey tools at the respective sites.

#### 2.4.1. Quantitative data collection

The demand-side assessment collected quantitative information through a structured, pilot-tested questionnaire in vernacular language. The information such as socio-demographic details, history of addiction and co-morbidities, patient's basic knowledge about the TB symptoms, social perception of TB patients, drug acceptance and adverse drug reactions, perception about the availed health services and perception of the healthcare workers and financial expenses were collected. The social perception was measured through a self-reported likert scale of five, mentioning agree to disagree. The additional care-seeking pathway section captured the sketch of all the events, i.e., detection of symptoms, seeking healthcare, first formal consultation, diagnosis, and treatment initiation to completion within the time frame. The delay assessment was captured from the care-seeking pathway section. Trained research assistants administered the survey tool to the selected TB patients in their households or at a place convenient to the patients in the vernacular language.

#### 2.4.2. Qualitative data collection

There were two sides to qualitative assessment. IDIs among TB patients aimed to explore the reasons for delay from the socio-cultural context, whereas among the system-side actors aimed to understand the delay reasons from the supply side. The trained researchers conducted the IDIs using the semi-structured interview guide in the vernacular language. The semi-structured interview guide for the system side actors was designed to capture barriers and challenges in implementing the NTEP, different aspects of the NTEP programs and the delays in the TB care cascade from the service provider's perspective. The semi-structured interview guide for the patients was designed with predecided themes of care-seeking pathways prior to the treatment initiation, delays within the care cascade and the challenges. Therefore, open-ended questions like details prior to the treatment initiation to treatment completion and different issues fronted during the various phase, TB medications, motivation, counseling details on drop-out, and reasons for the delay were included in the semi-structured interview guide.

### 2.5. Data analysis

The quantitative and qualitative data were handled independently. The below-mentioned procedures were followed for data analysis.

#### 2.5.1. Quantitative data analysis

Once the data collection was completed, data sets were imported from the application and validated, followed by data cleaning and analysis performed using statistical software STATA version 14.1. For the care-seeking pathway, the duration of delays across each care level was measured in the days from the care-seeking pathways section and then analyzed further. For the detailed analysis of the delays, patients were grouped into two categories i.e., pulmonary and extra-pulmonary TB patients and analyzed accordingly. Three categories of delay were estimated: (i) Delay in first formal consultation that led to confirmed diagnosis (onset of symptoms to first formal consultation); (ii) Diagnostic delay (consultation to confirmatory diagnosis) and (iii) Treatment delay (diagnosed to treatment initiation). The continuous variable of various delays was dichotomized using the cut-off values mentioned in the definitions for each phase of TB care cascade. The multivariate analysis was conducted using binomial logistic regression with delay as the outcome variable to understand the determinants for the delay in various stages of the care cascade. For the delay in the different cascade of TB, only three important variables i.e., type of TB (DSTB/DRTB), Age group (Adults/Pediatric) & place of services received (Public/Private Healthcare Facility) were included in the final analysis; whereas all the variables including other sociodemographic factors irrespective of statistical significance level were included in the multivariate analysis for the total delay.

The agree and disagree responses were averaged and reported for all the indicators under the social perception.

#### 2.5.2. Qualitative data analysis

The interviews were recorded, and verbatim notes were also taken during the interviews after the consent from the participants. The audios were transcribed and then translated into English. Manual descriptive content analysis was used to analyze the transcripts (22, 23). The decision on coding rules and quote generation were made by using standard procedures and in consensus with a specific focus on the phases of the delays (24). Both inductive and deductive codes were generated. To ensure that the results were a reflection of the data, the codes were related back to the quantitative data. The quotes were described as part of the qualitative findings and reported using Consolidated Criteria for Reporting Qualitative Research (25).

### 2.6. Definitions

Two sets of definitions were used, one for the delay in pulmonary and another for the delay in extra-pulmonary TB patients.

#### 2.6.1. Definitions of delay for pulmonary TB patients

**Figure 1:**
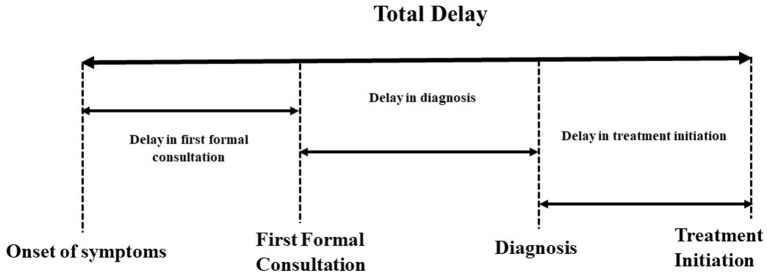
Delays in the various phases of TB care cascade.

- Delay in first formal consultation: The time interval between the onset of symptoms and the first formal consultation in TB patients. This was considered as more than 14 days as symptoms more than 14 days suggestive of TB as per the Standard for TB Care in India (STCI) guidelines (26).
- Delay in diagnosis: The time interval between the first formal consultation and diagnosis. This was considered as more than seven days (27).
- Delay in treatment initiation: The time interval between the diagnosis and treatment initiation. This was considered as more than seven days, as treatment should be initiated within seven days of diagnosis as per the NTEP guidelines (14).
- Total delay: A collective estimate of >28 days was used to define a cut-off value from the time interval between the onset of symptoms to initiation of anti-TB treatment, as depicted in Figure 1.

#### 2.6.2. Definition of delay for the extra-pulmonary TB patients

The median was considered as a cut-off value for the delay among the extra-pulmonary TB patients. Duration of days equal to or more than the median was considered the delay for the respective phases mentioned above. For the total delay also, a similar strategy was considered.

## 3. Results

### 3.1. Quantitative findings

A total of 990 TB patients were interviewed, as shown in Table 1, more than half of the patients (59.2%) were males, and 60.9% of the patients were 18–40 years old. Of all the patients, 15.8 % were illiterate, and only 11.6 % were graduates or had higher education. Almost one-fifth (21.2%) of the participants were daily laborers, 19.6% were housewives, and 24.9% were employed either owing business or with the organization. 31.5% of the patients reported having any addiction, and 12.3% reported co-morbidities. Almost 21.1% of the patients said their relatives and family members behave differently after being diagnosed with TB, and 10.1% said they have poor support from their family members.

**Table 1:** Characteristics of the TB patients participated in the study (*N* = 990).

**Variable**	**Total *n* (%)**
**Gender**	
Female	404 (40.8)
Male	586 (59.2)
**Age**	
≥17 years	86 (8.7)
18–40 years	603 (60.9)
41–60 years	238 (24.0)
>60 years	63 (6.4)
**Education**	
Illiterate	156 (15.8)
Never been to school but able to read and write	30 (3.0)
Primary	307 (31.0)
Secondary	215 (21.7)
Higher secondary	142 (14.3)
Graduate and Above	115 (11.6)
NA	25 (2.5)
**Occupation**	
Daily laborer	210 (21.2)
Housewives	194 (19.6)
Self employed	97 (9.8)
Student	135 (13.6)
Employed (private/ Govt.)	149 (15.1)
Other	39 (3.9)
NA	103 (10.4)
**Total family members**	
5 or < 5 members	658 (66.5)
>5 members	332 (33.5)
**BPL card**	345 (34.9)
**Marital status**	
Single	324 (32.7)
Married	620 (62.6)
Divorce/separated/widow	19 (1.9)
**Any kind of addiction**	312 (31.5)
**Any co-morbidity**	122 (12.3)
**Social perception**	
Poor family support	100 (10.1)
Feel isolated within the family	142 (14.3)
Their relatives and friends behave differently after being diagnosed with TB	209 (21.1)

NA, Not Applicable; BPL, Below poverty line.

As documented in Table 2, the mean duration between the onset of symptoms and formal consultation among the pulmonary patient was 30.9 (± 69.2) days, and the median was 10 days, the highest amongst all the phases of the TB care cascade. While in the extra-pulmonary patients, the mean duration between the onset of symptoms and formal consultation among the pulmonary patient was 40.4 (± 112.4) days, and the median was 12 days. The duration between formal consultation and the diagnosis was reported as 12.7 (±56.2) days in pulmonary and 14.0 (±50.0) in extra-pulmonary patients. The treatment was initiated in the next 7.4 (± 33.7) days in pulmonary and 7.1 (±20.7) in extra-pulmonary patients after diagnosis.

**Table 2:** Duration of the care-seeking at the various phase of the care cascade among surveyed TB patients in two Indian states Jharkhand, Gujarat during Jan–Jul 2021.

**Variable**	**Duration (in days) among pulmonary TB patients** **Median (IQR), mean ±SD** ***N*** **= 789 (%)**	**Duration (in days) among extra-pulmonary TB patients** **Median (IQR), mean ±SD** ***N*** **= 201 (%)**
Onset of symptoms to first formal consultation	10 (3–30), 30.9 ± 69.6	12 (5–31), 40.4 ± 112.4
First formal consultation to diagnosis	2 (0–5), 12.7 ± 56.2	2 (0–6), 14.0 ± 59.0
Treatment initiation	1 (0–3), 7.4 ± 33.7	1 (0–5), 7.1 ± 20.7
Total delay	19 (9–47), 51.0 ± 97.5	23 (12–58), 61.4 ± 127.3

Of the 990 patients, 789 (79.7) were pulmonary TB patients, and the remaining were extra-pulmonary. Most of the pulmonary TB patients (40.2%) reported a delay in initial formal consultation after the onset of symptoms, followed by a delay in diagnosis and treatment initiation. Almost 41% of the pulmonary patients reported total delay in the care cascade. In contrast, most of the extra-pulmonary TB patients (70.2%) reported a delay in treatment initiation, followed by a delay in diagnosis and first formal consultation. About half of the extra-pulmonary patients reported total delay in the care cascade, as indicated in Table 3.

**Table 3:** Delay at the various phase of the care cascade among surveyed TB patients in two Indian states Jharkhand, Gujarat during Jan–Jul 2021.

**Variable**	**Delay among pulmonary TB patients** ***N*** **= 789 (%)**	**Delay among extra pulmonary TB patients** ***N*** **= 201 (%)**
Delay in first formal consultation (onset of symptoms to consultation)	317 (40.2)	102 (50.8)
Delay in diagnosis (First formal consultation to diagnosis)	157 (19.9)	108 (53.7)
Delay in treatment initiation	109 (13.8)	141 (70.2)
Total delay (entire phase)	322 (40.8)	102 (50.8)
**Phase wise delay**		
Individuals with delay in any one phase	452 (52.3)	191 (95.0)
Individuals with delay in any two-phase	112 (14.2)	121 (60.2)
Individuals with delay in any three-phase	19 (2.4)	39 (19.4)

The multivariate logistic regression findings of the delays in different phases among pulmonary TB patients are depicted in Table 4. DR-TB patients were more likely to present with delay in treatment initiation (aOR = 3.49, 95%CI = 1.95–6.26) compared to DS-TB patients, found to be statistically significant. Pediatric patients were more likely to present with a delay in the diagnosis and treatment initiation (aOR = 1.71, 95%CI = 0.91–3.20; aOR = 1.46, 95%CI = 0.68–3.10) than the adults, while less likely to delay in the first formal consultation; however, found to be statistically non-significant. The patients availing services from the private health care facilities were more likely to have delay in first consultation (aOR = 1.07, 95%CI = 0.64–1.78) as compared to other phases and patients availing services from public health facilities (statistically non-significant). All the determinants for the extra-pulmonary TB were found to be statistically non-significant as indicated in Table 5. The pediatric patients were likelier to present delays in the first formal consultation and treatment initiation (aOR = 1.45, 95%CI = 0.68–3.10; aOR = 1.23, 95%CI = 0.53–2.84) than adults. DR-TB patients were more likely to present with delays in each phases i.e., first consultation (aOR = 2.14, 95%CI = 0.52–8.86), diagnosis (aOR = 7.51, 95%CI = 0.91–61.8) treatment initiation (aOR = 3.68, 95%CI = 0.45–30.23) compared to DS-TB patients.

**Table 4:** Analysis of the delay in the various care cascade phases amongst the various pulmonary TB patients (*N* = 789).

**Variable**		**Delay in the first consultation**	**Delay in diagnosis**	**Delay in treatment initiation**
		**aOR (95%CI)**	**aOR (95%CI)**	**aOR (95%CI)**
*R* square		0.0015	0.0071	0.0279
Constant		0.65 (0.55–0.77)	0.23 (0.19–0.28)	0.14 (0.11–0.18)
Type of TB	DS-TB (*n* = 728)	Reference	Reference	Reference
	DR-TB (*n* = 61)	1.38 (0.88– 2.34)	1.65 (0.91–2.99)	3.49 (1.95–6.26)
Age group	Adults (*n* = 736)	Reference	Reference	Reference
	Pediatric (*n* = 53)	0.92 (0.51–1.63)	1.71 (0.91–3.20)	1.46 (0.68–3.10)
Services received from	Public healthcare facility (*n* = 721)	Reference	Reference	Reference
	Private healthcare facility (*n* = 68)	1.07 (0.64–1.78)	0.79 (0.40–1.55)	0.66 (0.27–1.56)

aOR, Adjusted Odds Ratio; CI, Confidence Interval; TB, Tuberculosis; DS-TB, Drug Sensitive TB; DR-TB, Drug Resistant TB.

**Table 5:** Analysis of the delay in the various care cascade phases amongst the various extra-pulmonary TB patients (*N* = 201).

**Variable**		**Delay in the first consultation**	**Delay in diagnosis**	**Delay in treatment initiation**
		**aOR (95%CI)**	**aOR (95%CI)**	**aOR (95%CI)**
*R* square		0.0073	0.0239	0.0088
Constant		0.95 (0.69–1.32)	1.15 (0.83–1.58)	2.2 (1.56–3.12)
Type of TB	DS-TB (*n* = 192)	Reference	Reference	Reference
DR-TB (*n* = 9)	2.14 (0.52– 8.86)	7.51 (0.91–61.8)	3.68 (0.45–30.23)
Age group	Adults (*n* = 168)	Reference	Reference	Reference
Pediatric (*n* = 33)	1.45 (0.68–3.10)	0.99 (0.47–2.10)	1.23 (0.53–2.84)
Services received from	Public healthcare facility (*n* = 182)	Reference	Reference	Reference
Private healthcare facility (*n* = 19)	0.84 (0.32–2.17)	0.58 (0.22–1.54)	0.90 (0.32–2.50)

aOR, Adjusted Odds Ratio; CI, Confidence Interval; TB, Tuberculosis; DS-TB, Drug Sensitive TB; DR-TB, Drug Resistant TB.

It was observed that DR-TB patients were more likely to present with total delay in both pulmonary and extra-pulmonary patients, which was found to be statistically non-significant among the extra-pulmonary patients. In the pulmonary patients, addiction to any substance (aOR = 1.61, 95%CI = 1.12–2.34) and the presence of co-morbidity (aOR = 2.03, 95%CI = 1.28–3.21) were the common factors responsible for the total delay. Similarly, addiction (OR = 2.18, 95%CI = 0.85–5.59) and co-morbidity (aOR = 1.64, 95%CI = 0.59–4.61) were also identified as risk factors for delay in extra-pulmonary patients, however, found to be statistically non-significant. Unaware of monetary benefits (aOR = 1.85, 95%CI = 1.11–3.06) was also one of the contributing factors to the total delay among the pulmonary patients. As presented in detail in Table 6. Unawareness of free services for TB was also one of the risk factors in both categories, while illiteracy was only in pulmonary patients.

**Table 6:** Factors contributing to the total delay in various groups of TB patients.

**Variable**		**Pulmonary (*n* = 789)**	**Extra pulmonary (*n* = 201)**
		**aOR (95%CI)**	**aOR (95%CI)**
*R* square		0.0507	0.0566
Constant		0.68 (0.38–1.22)	0.73 (0.27–1.98)
Type of TB	DS-TB	Reference	Reference
	DR-TB	3.36 (1.91–5.93)	4.00 (0.77–20.8)
Age group	Adult	Reference	Reference
	Pediatric	1.35 (0.70–2.60)	2.07 (0.74–5.73)
From where services availed	Public healthcare facilities	Reference	Reference
	Private healthcare facilities	1.34 (0.79–2.29)	1.12 (0.41–3.11)
Age^*^		0.99 (0.97–1.0)	0.99 (0.97–1.03)
Total family members^*^		1.02 (0.95–1.08)	1.0 (0.99–1.00)
Total family income^*^		1.0 (0.99–1.00)	1.0 (0.99–1.00)
Gender	Female	Reference	Reference
	Male	1.03 (0.72–1.48)	0.96 (0.51–1.83)
Education	Literate	Reference	Reference
	Illiterate	1.06 (0.73–1.54)	0.91 (0.33–2.46)
Marital status	Single	Reference	-
	Married	1.15 (0.78–1.72)	-
	Divorced/seperated/widow	1.47 (0.49–4.42)	-
Free services for the TB	Aware	Reference	Reference
	Unaware	1.28 (0.75–2.20)	1.64 (0.62–4.39)
Monetory benefit for treatment	Aware	Reference	Reference
	Unaware	1.85 (1.11–3.06)	0.83 (0.6–2.66)
Any addiction	Not present	Reference	Reference
	Present	1.61 (1.12–2.34)	2.18 (0.85–5.59)
Any comorbidity	Not present	Reference	Reference
	Present	2.03 (1.28–3.21)	1.64 (0.59–4.61)
Family support	Good	Reference	Reference
	Poor	0.59 (0.36–0.96)	0.85 (0.25–2.87)
Feel isolated within family	Feel not isolated	Reference	Reference
Feel isolated	0.86 (0.54–1.38)	2.53 (0.79–8.17)
Their relatives and friends behave differently	Behavior not changed	Reference	Reference
Changed behavior	0.83 (0.55–1.24)	0.34 (0.13–0.92)

aOR, Adjusted Odds Ratio; CI, Confidence Interval; TB, Tuberculosis; DS-TB, Drug Sensitive TB; DR-TB, Drug Resistant TB.

* continuous variables.

### 3.2. Qualitative findings

#### 3.2.1. System side actors' perception

The respondents reported that the unawareness among the community and stigma as one of the important factors responsible for the delay in the first formal consultation. Both system and demand side factors emerged as the reasons for the delay in the diagnosis. The overburden of the staff with work and negligence from the providers were reasons for the delay in the diagnosis. The stigma, addiction, lack of awareness and financial constraints contribute to the delay in the diagnosis. While for the treatment initiation, mainly the patients' behavior and lack of knowledge on the importance of treatment emerged as one of the factors responsible for the delay. Moreover, the delay in the first formal consultation was directly attributed to the missing TB cases by the system, the active case findings were not qualitatively assured or supervised and monitored, and a few gaps in contact tracing because of the migrant population. In the diagnostic cascade, the delay was attributed majorly to the poor infrastructure, quality of sputum samples submitted by patients, sample collection to transportation, and neglected referrals of TB comorbid cases. In the treatment cascade, the delay was attributed to the adverse drug reaction and its management and inadequate counseling by the healthcare staff. The major quotes are indicated below-

“*There are few cases of the same (diagnosed but not put on treatment). Many are transfer outs and not recorded. Self-denial and doctor- hopping are common reasons.”* (State TB Training & Demonstration Center Director).

“*It is a fact that wherever we go(national level or local level) patients go for multiple consultations. If the person is educated, he will go for investigation but here it is a tribal area, so less awareness about TB…..”* (Senior Treatment Supervisor).

“*……Social stigma, Unawareness among the community, Financial constrain are among other prime factors in this rural part of the state.”* (Medical Officer).

“*delay in diagnosis is from the system side, due to workload not able to perform CBNAAT on time.”* (Senior TB Laboratory Supervisor).

“*…….2–3 days delay in turnaround time for CBNAAT, due to higher workload”* (Senior TB Laboratory Supervisor).

“*(Maximum delay) in diagnosis sir… due to poor infrastructure... there is no lacking on the part of patients…patients are coming at right place only but because of poor infrastructure, we are able to provide timely services…”* (Senior Treatment Supervisor)

“*There is delay. There is huge negligence in private sectors. Private doctors initiate general treatment and they do not keep TB in their differential diagnosis……”*. (Medical Officer).

“*A bit of delay in diagnosis is the from system side issues. Secondly due to lack of awareness patient could not reach to us… there are the two main reason in TB”* (Medical Officer).

“*Patient does not feel the importance of getting diagnosed, so they refuse sometimes. Social stigma. Alcoholism.”* (Laboratory Technician).

“*Main reason for the delay in diagnosis is that people are alcoholic, financial constraint (transportation) and lack of awareness and importance of the disease.”* (TB health visitor).

“*Some patients (TB confirmed) refuse to take medicine because of addiction (smoking/alcohol). Takes time to convince them.”* (Laboratory Technician).

“*Patient having no knowledge on importance of treatment. Even patients having MDR TB along with comorbid condition refuses for treatment management at DTC.”* (Medical Officer).

#### 3.2.2. Demand side perception

The patients shared that the lack of knowledge regarding the TB symptoms and addiction were the major reasons responsible for the delay in care seeking at various phases of the care cascade. The fear of isolation, health-seeking behavior and the perceived severity of the symptoms also contribute to the delay. Moreover, the delays in the first formal consultation phase were attributed to the ease of access to the traditional healers, patient literacy about TB care, and health-seeking behavior and attitude of the respective patients. During the diagnostic cascade, the delay was attributed to the stigma related to the TB diagnosis and fear, patients were from remote areas and traveled only for consultation. In the treatment care cascade, the delay was attributed to the potential discrimination for taking TB drugs for a long duration by family/ community, poverty leading to an inability for physical access to the services, migration as part of the livelihood, adverse drug reactions potentially prolong use of substance abuse and addictions and multiple reasons for treatment interruptions.

“*I thought there was some delay in treatment initiation because at that time I was not aware regarding TB symptoms…..”* (DS-TB patient).

“*…..Who take medicines, it is better to have alcohol rather than medicines”* (DR-TB patient).

“*Few people forced (indicated surrounded neighbors) that if my symptoms are TB, then they will not mingle with me…….So, I was afraid”* (DS TB patient).

“*Initially, I thought the symptoms are seasonal, so brough a few medicines from one of the nearest doctor (informal provider). It was good for few days. When symptoms again aggravated, I went to the Govt. hospital and there they said it's TB……”* (EP TB patients).

“*I took medicines for cough and cold over 3 months, but symptoms didn't subside”* (Extra-pulmonary TB patient).

“*…and chemist also said that this is the normal cough, and having cough in this season is quite normal so, it will be fine after few days of medication”* (DS-TB patient).

“*What does a layman do, when (s)he develops cold/cough, then simply go to a chemist and take medicines; I did the same”* (DS TB patient)“*…almost 5 months taken for proper treatment initiation. Initially went to the informal provider where almost Rs. 2,000 spent then went to private doctors where tests were done and medicine given but not got relief…….”* (MDR-TB patient).

## 4. Discussion

This study aimed to assess the delay among TB patients, which is an important factor contributing to the successful treatment outcome. The findings of this study revealed a substantial delay in care-seeking at the various phases of the care cascade in TB patients. Our study found that the delay in the first formal consultation of 41% is the highest among all three delays across the phases of the care cascade in pulmonary patients. This prevalence rate of delay in care-seeking is quite similar to what was found in studies from Ethiopia and South Africa (28, 29). However, studies from various states of India reported a delay in care-seeking ranging from 27 to 74% (14, 21, 27, 30). In contrast, delay in treatment initiation is highest in extra-pulmonary patients, and almost half of the patients reported total delay. Patients with DR-TB are more prone to report the total delay in the care cascade among the pulmonary patiets. One of the Indian studies also reports that the median onset of symptoms to first consultation and first consultation to diagnosis was higher in multidrug-resistant (MDR) patients than the non-MDR (31).

The findings from the systematic review from India states the median patient delay (i.e., delay in first formal consultation), diagnostic delay and treatment delay of 18.4, 31.0, and 2.5 days, respectively (13); this median duration is higher than the duration we found in this study. One of the possible reasons for these variations in median and prevalence could be that delay is defined differently in each study.

The addiction to any substance among TB patients emerged as one of the contributing factors to total delay in pulmonary TB patients. This was also found in other studies (14, 17, 21, 32) conducted in various categories of TB patients. Various studies from different parts of the country stated that literacy level (32), financial constraints (14, 15, 17, 30, 33, 34), myths / wrong beliefs (14, 15, 17, 21, 30, 33–36), knowledge regarding availability of the services (15, 32), contribute to the delay at various phases of the care cascade. This study also reports the unawareness among the patients regarding the services available as one of the risk factors for total delay among pulmonary patients. This highlights the importance of creating awareness among the community regarding the disease and the services and benefits available. Another finding is that illiteracy is one of the risk factors for delay in pulmonary patients (although statistically non-significant, this was evident in qualitative findings).

In a few studies, social stigma, discrimination (34, 36, 37) and lack of family support (17) were identified as the contributing factors to delay in care-seeking. However, in particular, in this study, we did not find any significant difference in family support, discrimination and stigma as risk factors for the delay; however, in qualitative, it was prompted.

Understanding delay and its factors will minimize patient loss across the TB care cascade, as evidenced in the literature (38, 39). Future research on a similar topic with various TB patients across India is recommended to understand the divergent delay and its associated contextual factors. There is a lack of evidence in extra-pulmonary TB patients; however, few international studies are available (40). Hence, the delayed assessment should comprise extra-pulmonary TB patients distinctly different from the pulmonary TB care-seeking pathways recommended for contextual understanding. Documenting the delay attributes across the different geographical contexts is important, as both the system and demand side factors vary significantly. A comprehensive strategy addressing all these factors is recommended within the national TB elimination program to minimize the delays in the TB care cascade and ultimately achieve the larger goal of *End TB strategy*.

One of the limitations of this study is that all the data was collected retrospectively, so patients may fail to remember the exact date for the events of the care cascade. Therefore, there is a chance of recall bias. However, all the research teams involved in the data collection process trained thoroughly to get the most accurate details from the patients. Secondly, we could not include the missing cases that were not notified under the nikshay. Third, there were no such thumb rules to include minimum number of TB patients per each categories such as DR-TB and pediatric TB. We have intended to capture minimum of 30 patients and a diverse representation to run the statistical analysis. Fourth, we were able to recruit only 10 TB patients as part of the qualitative enquiry. In an ideal scenario for qualitative data collection, data should be collected till saturation; however, due to the project timeline, we were not in a position to continue the data collection, which is considered one of the limitations of the demand side qualitative assessment.

## 5. Conclusion

In this cross-sectional study, more than half (52.3%) of pulmonary TB patients reported a delay in care-seeking during any phase of the care cascade. While in the extra-pulmonary patients, it is even higher than that. Almost 41% of the pulmonary and 51% of the extra-pulmonary patients had reported total delay in the care cascade. Out of all three delays at the various phases of the care cascade, delay in initial formal consultation after the onset of the symptoms is most common, followed by a delay in diagnosis and treatment initiation in pulmonary patients. While in extra-pulmonary patients, delay in treatment initiation is most common, followed by the diagnosis and first formal consultation. DR-TB patients are more prone to total delay and delay in the treatment initiation among pulmonary patients; however, a similar observation was statistically non-significant among the extra-pulmonary patients. Factors like awareness regarding monetary benefits available for TB patients, addiction, and co-morbidity contribute significantly to the total delay among pulmonary TB patients. There were a few systems-side factors, such as inadequate case findings in the first formal consultation, poor infrastructure in the diagnostic cascade and inadequate adverse drug reaction management in the treatment cascade, among other attributes to the delay. The TB care cascade delay is multi-dimensional, as indicated by both the system and demand side attributes having their own contribution. Therefore, the balance between service delivery and socio-cultural context needs strategies to address the delays in the TB care cascade.

## Data availability statement

The raw data supporting the conclusions of this article will be made available by the authors, without undue reservation.

## Ethics statement

The studies involving human participants were reviewed and approved by Institutional Ethics Committee (IEC) of the Indian Institute of Public Health Gandhinagar (IIPHG), India. The patients/participants provided their written informed consent to participate in this study.

## Author contributions

SY, HS, SS, and DS participated in the conception and design of the study protocol. SS, AS, TP, YP, and DS involved in project administration. SY, HS, PB, and JP collected the field data and conducted the interviews. SY and PB analyzed the data. PB drafted the first draft of the paper. SY, HS, SS, AS, TP, YP, and DS critically reviewed the paper. All authors contributed equally to the development of this study and read and approved the final manuscript.
