# Supplementary material for: Why and where?—Delay in Tuberculosis care cascade: A cross-sectional assessment in two Indian states, Jharkhand, Gujarat
Source: Front Public Health. 2023 Jan 27;11:1015024. doi: 10.3389/fpubh.2023.1015024 (PMC9911525; doi:10.3389/fpubh.2023.1015024)
Supplement: Supplementary file 1 [file Table_1.pdf]

Annexure Table-1. Delay in the various phases of the care cascade amongst the pulmonary TB patients (N=789)

**Variable**		**Delay in the first consultation**			**Delay in diagnosis**			**Delay in treatment initiation**		
		**No (n=472)**	**Yes (n=317)**	***c*OR (95%CI)**	**No (n=632)**	**Yes (n=157)**	***c*OR (95%CI)**	**No (n=680)**	**Yes (n=109)**	***c*OR (95%CI)**
**Type of TB**	DS-TB (n=728)	440 (93.2)	288 (90.9)	Reference	588 (93.0)	140 (89.2)	Reference	639 (94.0)	89 (81.7)	Reference
	DR-TB (n=61)	32 (6.8)	29 (9.2)	1.38 (0.82 – 2.33)	44 (7.0)	17 (10.8)	1.62 (0.90 – 2.93)	41 (6.0)	20 (18.4)	3.50 (1.96 – 6.25)
**Age group**	Adults (n=736)	439 (93.0)	297 (93.7)	Reference	594 (94.0)	142 (90.5)	Reference	636 (93.5)	100 (91.7)	Reference
	Pediatric (n=53)	33 (7.0)	20 (6.3)	0.90 (0.50 – 1.59)	38 (6.0)	15 (9.6)	1.65 (0.88 – 3.09)	44 (6.5)	9 (8.3)	1.30 (0.62 – 2.75)
**Services received from**	Public Healthcare Facility (n=721)	432 (91.5)	289 (91.2)	Reference	575 (91.0)	146 (93.0)	Reference	618 (90.9)	103 (94.5)	Reference
	Private Healthcare Facility (n=68)	40 (8.5)	28 (8.8)	1.05 (0.63 – 1.73)	57 (9.0)	11 (7.0)	0.76 (0.39 – 1.49)	62 (9.1)	6 (5.5)	0.58 (0.24 – 1.34)

cOR: Crude Odds Ratio; CI: Confidence Interval; TB: Tuberculosis; DS-TB: Drug Sensitive TB; DR-TB: Drug Resistant TB

Annexure Table 2. Delay in the various phases of the care cascade amongst the extra pulmonary TB patients (N=201)

**Variable**		**Delay in the first consultation**			**Delay in diagnosis**			**Delay in treatment initiation**		
		**No (n=99)**	**Yes (n=102)**	***c*OR (95%CI)**	**No (n=93)**	**Yes (n=108)**	***c*OR (95%CI)**	**No (n=60)**	**Yes (n=141)**	***c*OR (95%CI)**
**Type of TB**	DS-TB (n=192)	96 (97.0)	96 (94.1)	Reference	92 (98.9)	100 (92.6)	Reference	59 (98.3)	133 (94.3)	Reference
	DR-TB (n=9)	3 (3.0)	6 (5.9)	2.0 (0.49 - 8.23)	1 (1.1)	8 (7.4)	7.36 (0.90 – 60.0)	1 (1.7)	8 (5.7)	3.55 (0.43 – 29.0)
**Age group**	Adults (n=168)	85 (85.9)	83 (81.4)	Reference	77 (82.8)	91 (84.3)	Reference	51 (85.0)	117 (83.0)	Reference
	Pediatric (n=33)	14 (14.1)	19 (18.6)	1.38 (0.65 - 2.95)	16 (17.2)	17 (15.7)	0.90 (0.43 – 1.90)	9 (51.0)	24 (17.0)	1.16 (0.50 – 2.68)
**Services received from**	Public Healthcare Facility (n=182)	89 (89.9)	93 (91.2)	Reference	82 (88.2)	100 (92.6)	Reference	54 (90.0)	128 (90.8)	Reference
	Private Healthcare Facility (n=19)	10 (10.1)	9 (8.8)	0.86 (0.33 - 2.22)	11 (11.8)	8 (7.4)	0.60 (0.23 – 1.55)	6 (10.0)	13 (9.2)	0.91 (0.33 - 2.53)

cOR: Crude Odds Ratio; CI: Confidence Interval; TB: Tuberculosis; DS-TB: Drug Sensitive TB; DR-TB: Drug Resistant TB

Annexure Table-3. Factors contributing to the total delay among the pulmonary of TB patients (N=789)

Varibales		No Delay (n=467)	Delay (n=322)	*c*OR (95%CI)
**Type of TB**	DS-TB	445 (95.3)	283 (87.9)	Reference
	DR-TB	22 (4.7)	39 (12.1)	2.78 (1.62 – 4.80)
**Age Group**	Adult	86 (86.9)	82 (80.4)	Reference
	Pediatric	13 (13.1)	20 (19.6)	1.32 (0.75 – 2.30)
**From where services availed**	Public healthcare facilities	90 (90.9)	92 (90.2)	Reference
	Private healthcare facilities	9 (9.1)	10 (9.8)	1.09 (0.66-1.79)
**Age***				0.99 (0.98 – 1.0)
**Total Family Members***				1.03 (0.96 – 1.09)
**Total Family Income***				1.0 (0.99 – 1.00)
**Gender**	Female	184 (39.4)	114 (35.4)	Reference
	Male	283 (60.6)	208 (64.4)	1.19 (0.88 – 1.59)
**Education**	Literate	359 (76.9)	242 (75.2)	Reference
	Illiterate	108 (23.1)	80 (24.8)	1.09 (0.79 – 1.53)
**Marital Status**	Single	152 (32.6)	107 (33.2)	Reference

	Married	306 (65.5)	207 (64.3)	0.96 (0.71 – 1.30)
	Divorced/seperated/widow	9 (1.9)	8 (2.5)	1.26 (0.47 – 3.38)
**Free services for the TB**	Aware	427 (91.4)	281 (87.3)	Reference
	Unaware	40 (8.6)	41 (12.7)	1.56 (0.98 – 2.47)
**Monetory benefit for treatment**	Aware	425 (91.0)	270 (83.9)	Reference
	Unaware	42 (9.0)	52 (16.2)	1.94 (1.26 – 3.00)
**Any Addiction**	Not present	319 (68.3)	191 (59.3)	Reference
	Present	148 (31.7)	131 (40.7)	1.48 (1.10 – 1.99)
**Any comorbidity**	Not Present	419 (89.7)	271 (84.2)	Reference
	Present	48 (10.3)	51 (15.8)	1.64 (1.08 – 2.51)
**Family support**	Good	410 (87.8)	291 (90.4)	Reference
	Poor	57 (12.2)	31 (9.6)	0.77 (0.48 – 1.22)
**Feel isolated within family**	Feel not Isolated	388 (83.1)	276 (85.7)	Reference
	Feel Isolated	79 (16.9)	46 914.3)	0.82 (0.55 – 1.21)
**Their relatives and friends behave differently**	Behaviour not Changed	354 (75.8)	252 (78.3)	Reference
	Changed Behaviour	113 (24.2)	70 (21.7)	0.87 (0.62-1.24)

cOR: Crude Odds Ratio; CI: Confidence Interval; TB: Tuberculosis; DS-TB: Drug Sensitive TB; DR-TB: Drug Resistant TB

Annexure Table-4. Factors contributing to the total delay in extra- pulmonary of TB patients (N=201)

Variables		No Delay (n=99)	Delay (n=102)	*c*OR (95%CI)
**Type of TB**	DS-TB	97 (98.0)	95 (93.1)	Reference
	DR-TB	2 (2.0)	7 (6.9)	3.57 (0.72 - 17.64)
**Age Group**	Adult	86 (86.9)	82 (80.4)	Reference
	Pediatric	13 (13.1)	20 (19.6)	1.61 (0.75 – 3.45)
**From where services availed**	Public healthcare facilities	90 (90.9)	92 (90.2)	Reference
	Private healthcare facilities	9 (9.1)	10 (9.8)	1.09 (0.42 – 2.80)
**Age***				1.00 (0.98 – 1.01)
**Total Family Members***				1.00 (0.99 – 1.00)
**Total Family Income***				1.00 (0.99 – 1.00)
**Gender**	Female	55 (55.6)	51 (50.0)	Reference
	Male	44 (44.4)	51 (50.0)	1.25 (0.72 – 2.17)
**Education**	Literate	88 (88.9)	90 (88.2)	Reference
	Illiterate	11 (11.1)	12 (11.8)	1.07 (0.45 – 2.54)
	Aware	90 (90.9)	89 (87.3)	Reference

**Free services for the TB**	Unaware	9 (9.1)	13 (12.8)	1.46 (0.59 – 3.59)
**Monetory benefit for treatment**	Aware	92 (92.9)	94 (92.2)	Reference
	Unaware	7 (7.1)	8 (7.8)	1.12 (0.39 – 3.21)
**Any Addiction**	Not present	86 (86.9)	82 (80.4)	Reference
	Present	13 (13.1)	20 (19.6)	1.61 (0.75 – 3.45)
**Any comorbidity**	Not Present	88 (88.9)	90 (88.2)	Reference
	Present	11 (11.1)	12 (11.8)	1.07 (0.45 – 2.54)
**Family support**	Good	93 (93.9)	96 (94.1)	Reference
	Poor	6 (6.1)	6 (5.9)	0.97 (0.30 – 3.11)
**Feel isolated within family**	Feel not Isolated	92 (92.9)	92 (90.2)	Reference
	Feel Isolated	7 (7.1)	10 (9.8)	1.43 (0.52 – 3.91)
**Their relatives and friends behave differently**	Behaviour not Changed	83 (83.8)	92 (90.2)	Reference
	Changed Behaviour	16 (16.2)	10 (9.8)	0.56 (0.24 – 1.31)

cOR: Crude Odds Ratio; CI: Confidence Interval; TB: Tuberculosis; DS-TB: Drug Sensitive TB; DR-TB: Drug Resistant TB
